# Interactive effect of leader ethicality and competency on Chinese customs officers’ organizational citizenship behaviors

**DOI:** 10.3389/fpsyg.2023.1152608

**Published:** 2023-11-24

**Authors:** Zhenbang Fang, Min Hua, Yuanjie Bao, Qi Sun

**Affiliations:** ^1^School of Public Administration and Policy, Renmin University of China, Beijing, China; ^2^Tianjin Customs, Tianjin, China

**Keywords:** ethical leadership, leader competency, organizational citizenship behavior, trust in leader, work engagement

## Abstract

The dual qualities of an effective leader—ethicality and competency—have long been identified but seldom empirically examined. Using survey data from 329 Chinese customs officers collected in December 2022, this study investigates whether ethical leadership influences customs officers’ organizational citizenship behaviors indirectly through work engagement and trust in leader. Following the interactive approach, we further postulate that leader competency can accentuate these indirect relationships. Mplus 8.3, SPSS 26.0 and Hayes’ PROCESS macro for SPSS were used to conduct statistical analyses including descriptive statistical analysis, correlation analysis, common method deviation analysis, confirmatory factor analysis, and regression analysis. The results reveal that work engagement and trust in leader act as mediators in the ethical leadership and organizational citizenship behaviors relationship. Moreover, these indirect relationships are stronger when customs officers perceive their leaders are more competent. Theoretical and practical implications are discussed.

## 1 Introduction

Scandals on leader morality in private and public sectors worldwide have drawn widespread attention to the ethical issues concerning leaders (Den Hartog, 2015), thus rendering leader ethicality a prominent field of research (Ng and Feldman, 2015; Hoch et al., 2018). Leader ethicality has been associated with various attitudinal, behavioral, and wellbeing outcomes of followers (Mostafa et al., 2020; Mohsin et al., 2021; Li et al., 2022; Mansour et al., 2022; Lin et al., 2023). In fact, both private and public organizations try to implement selection and development devices to benefit from this important leadership approach (Kim, 2023).

Although ethical leadership has been examined in the public sector and have revealed similar or even more profound influences (Bedi et al., 2015), some issues remain underexplored. Firstly, studies have long claimed the importance of both leader ethicality and competency in the public sector (Van Wart, 2011, 2013; Bundgaard et al., 2021). Specifically, public organizations depend on professional bureaucrats who not only serve in an ethical and professional way but also might require their leaders to display such combination of ethicality and competency. In other words, the influence of ethical leadership in professional public organizations might be conditioned upon whether followers deem their leaders as competent (Hollander, 1978). However, whether and how leader ethicality and competency interactively influence the behavior of professional public employees remain underexplored. Secondly, the mechanisms by which ethical leadership affects public organizations remain unclear (Mostafa and Abed El-Motalib, 2018; Belle and Cantarelli, 2019). Behavioral public administration studies have predominantly applied a motivational approach to examine the influence of ethical leadership on followers’ prosocial orientation or public service motivation, while the way public employees approach work and work relationship under the influence of ethical leadership is not given sufficient attention. Thirdly, the outcomes of ethical leadership in public sector mainly focus on ethical/unethical behaviors such as invincibility (Young et al., 2021) or corruption (Bashir and Hassan, 2020). Nevertheless, the effects on proactive behaviors, including voice (Hassan, 2015) and organizational citizenship behaviors, have rarely been investigated (Kacmar and Bachrach, 2011; Potipiroon and Faerman, 2016).

This study recognizes the importance of ethical leadership in professional public organizations and the limitations of the extant literature, and examines how ethical leadership influences the organizational citizenship behavior (OCB) of public employees. Specifically, the research question of this study is: is ethical leadership related to OCB? If so, does work engagement and trust in leader mediate this relationship? Can leader competency moderate the influence of ethical leadership such that its influences are strengthened? As such, the aim of this study to examine the effectiveness of the duality of ethicality and competency of leaders among public employees. This study uses a sample of Chinese customs officers and focuses on the mediating role of work engagement and trust in the leader and the moderating role of leader competency. Based on survey data from 329 Chinese customs officers collected in the December of 2022, we used hierarchical regression and bootstrapping methods to test the hypothesized moderated mediation model. The results are consistent with our hypotheses.

This study contributes to the literature in three ways. First, we ascertain whether ethical leadership is related to the OCB of public employees that is not only proactive in nature but also goes beyond the current emphasis on ethical-oriented behavior within the behavioral public administration research. Doing so extends the nomological network of ethical leadership in public administration by emphasizing the cooperative nature of public service provision. Second, we delineate the effect mechanism of ethical leadership by simultaneously incorporating the important mediating roles of work engagement and trust in the leader. That is, by unraveling and juxtaposing both work engagement (representing followers’ relationships with their work) and trust in leader (representing followers’ relationships with their leader), we simultaneously include the most important relationships that ethical leaders can build for their followers through displaying ethical behaviors. By understanding the influence of ethical leadership on followers’ relationships with both the work itself and the working relationship, the knowledge on ethical leadership’s motivational function can be extended. Third, by exploring the moderating role of leader competency, we not only clarify the boundary condition of ethical leadership from a leader competency perspective (Bedi et al., 2015) but also highlight the long-standing but seldom examined claim of the duality of leader ethicality and competency in public administration, whereby both prosocial and professional characteristics are emphasized from an interactive approach.

The structure of this article is as follows. The next section discusses prior research on ethical leadership in the context of public sector organizations, highlights its research gaps, and develops hypotheses based on social learning, social exchange, and interactive psychology theories. Afterwards, we elaborate on the participants and procedures of the survey, as well as the measures that were used. Results are then presented, followed by the theoretical and practical implications, as well as the discussions of the limitations and future directions.

## 2 Theoretical framework and hypotheses

### 2.1 Ethical leadership and organizational citizenship behaviors in public administration

Studies have established the importance of ethicality in public administration (Niskanen, 1971; Perry and Wise, 1990) and have suggested that the provision of public service should be conducted in a moral and prosocial manner (Brehm and Gates, 1997; Frey and Palacios-Huerta, 1997). Public service ethos, motivation, and values have been emphasized in the research on behavioral public administration (Perry and Vandenabeele, 2015). Moreover, given that the quality of leadership influences the way public employees behave (Van Wart, 2003, 2012, 2013; Bundgaard et al., 2021), the effectiveness of ethical leadership has become an important topic (Erickson, 2006; Hassan et al., 2014; Weinberg, 2014; Belle and Cantarelli, 2019; Young et al., 2021). Ethical leadership is defined as “the demonstration of normatively appropriate conduct through personal and interpersonal relationships, and the promotion of such conduct to followers through two-way communication, reinforcement, and decision-making” (Brown et al., 2005, p. 120), and investigations have found that it leads to reduced unethical behaviors (Belle and Cantarelli, 2019; Bashir and Hassan, 2020; Young et al., 2021; Kim, 2023), improved job satisfaction (Moon and Jung, 2018), and organizational commitment (Hassan et al., 2013, 2014) among public employees. In addition, a recent meta-analysis reported that ethical leadership is more influential on followers working in the public sector than those in the private sector (Bedi et al., 2015), which highlights that ethical leadership is a prominent and promising field of investigation in public administration.

Although the influence of ethical leadership on public employees’ proactive behaviors is increasingly investigated, the examined outcomes mainly focus on ethicality-related behaviors such as willingness to report ethical problems (Wright et al., 2016), while OCB is not paid much attention (Potipiroon and Faerman, 2016; Mozumder, 2018). OCB refers to “individual behavior that is discretionary, not directly or explicitly recognized by the formal reward system, and that in the aggregate promotes the effective functioning of the organization” (Organ, 1988, p. 4). It is highly important in public service because it fits well with the prosocial and collaborative nature of public service and goes beyond the sole monetary consideration of formal reward system (Molines et al., 2022). Therefore, researchers have tried to locate the antecedents of OCB within public organizations (Campbell and Im, 2016), especially from a leadership perspective (Ritz et al., 2014; Chen et al., 2023).

We conjecture that ethical leadership leads to public employee’s OCB for two reasons. First, ethical leaders preach moral values, instill righteous and moral ideals, behave consistently with their words, act in prosocial ways (Brown, 2007; Treviño and Brown, 2007; Fehr et al., 2015). Consequently, followers learn what is emphasized, considered right, and valued in the organization, and thus, develop a moral and prosocial orientation (Brown et al., 2005). According to social learning theory, followers treat ethical leaders as credible role models and act in prosocial and proactive ways (Brown et al., 2005; Kaptein et al., 2005), thus display more OCB. Second, the care and concern provided by ethical leaders are deemed important social resources by followers (Brown and Treviño, 2006; Bedi et al., 2015). According to social exchange theory, to reciprocate these kind treatments from the leaders, followers develop OCBs that benefit their organization and colleagues (Blau, 1964; Cropanzano and Mitchell, 2005). Therefore, we propose the following hypothesis.

*Hypothesis 1: Ethical leadership is positively related to public employees’ OCB.*

### 2.2 The mediating roles of work engagement and trust in leader

Given the limited attention paid to the mechanisms by which ethical leadership influences public administration beyond prosocial motivation, we focus on how work engagement and trust in the leader might simultaneously mediate between ethical leadership and OCB. In this regard, we answer the important question of how ethical leadership motivates OCB through public employees’ relationships with the work itself and with their leaders. We choose work engagement and trust in leader as parallel mediators simultaneously mediating the influence of ethical leadership because the former represent followers’ relationship with the work itself while the later represent the relationship with their leaders. That is, by utilizing work engagement and trust in leader, we would get a more comprehensive and nuanced understanding on the effect mechanism of ethical leadership, extending extant knowledge on the motivational influence of ethical leadership in the public domain.

Work engagement defined as “a positive, fulfilling, work-related state of mind that is characterized by vigor, dedication, and absorption” (Schaufeli et al., 2002, p. 74). It reflects employees’ involvement with and enthusiasm for work (Breevaart et al., 2016). On the one hand, ethical leaders encourage the feeling of engagement among followers. According to social learning theory, learning processes characterized by observational learning, imitation, and identification (Davis and Luthans, 1980; Bandura, 1991) are fertile under the influence of ethical leaders (Brown et al., 2005). In this scenario, followers learn how ethical leaders approach their work, become more open to their influence, and identify with their values and ideals. Consequently, the work becomes a way of self-expression and the followers become engaged. According to social exchange theory, the care and concern expressed by ethical leaders become important job resources. According to the job demand–resource model of engagement (Bakker and Demerouti, 2014), the inflow of resources induced by ethical leaders stimulates the enthusiasm of followers for work to reciprocate the organization. Recent empirical evidence also suggests the influence of ethical leadership on public employees’ engagement (Lu and Guy, 2014; Asif et al., 2019; Mostafa et al., 2020; Zahari and Kaliannan, 2023). On the other hand, engagement is a strong predictor of OCB (Xu et al., 2019; Stein et al., 2021). When engaged, an individual has more resources to develop and expand behaviors outside of formal roles to contribute to their organization and colleagues. Recent empirical evidence supports this relationship (Tian et al., 2021; Zhang and Farndale, 2022).

Trust in the leader reflects an individual’s positive expectations of the competence, reliability, and benevolence of their leaders (Dirks and Ferrin, 2002; Burke et al., 2007). Essentially, trust in the leader refers to an individual’s “psychological state comprising the intention to accept vulnerability based upon positive expectations of the intentions or behavior” of the leader (Rousseau et al., 1998). This definition suggests that ethical leaders should earn the trust of followers because they act in open, fair, and honest ways. Behavioral integrity and consistency render a psychologically safe environment. When followers believe that their leaders act in an ethical manner, they become more confident that such leaders will not manipulate them, take credit for their contributions, or take advantage of them. Moreover, they tend to reciprocate this high-quality relationship with more OCB, which is consistent with the argument of social exchange theory. Leaders are treated as the representative of the organization, and an important way to express and repay the favorable treatment from an ethical leader is to go beyond the formal job requirements to display OCB. In fact, studies have demonstrated the influence of trust in the leader on OCB (Zhu et al., 2013; Lu, 2014). Hence, we propose the following hypothesis.

*Hypothesis 2: Work engagement (hypothesis 2a) and trust in the leader (hypothesis 2b) mediates the positive relationship between ethical leadership and OCB.*

### 2.3 The moderating role of leader competency

Although important, ethicality is only one of the ways leaders can influence their followers. Being an ethical leader does not guarantee that one is competent at setting relevant goals, making good decisions, arranging procedures and regulations, dividing work and resources, and motiving and controlling followers. In this sense, the influence of ethical leadership might depend on the level of leader competency. According to interactional psychology, the influence of ethical leadership might be contingent upon other important leadership characteristics, leader competency in our case. Leader competency refers to the knowledge, skills, experience, and wisdom required to succeed as a leader (Wei et al., 2018; Mao et al., 2019; Cai et al., 2023). It is considered an important component of leader effectiveness (Hollander, 1978). Cognitive psychology has long proven that people are more strongly influenced by the behaviors of competent leaders (Justis et al., 1978; Price and Garland, 1981; Podsakoff et al., 1983; Fiske et al., 2007). Recent developments in leadership research also point out the crucial role of leader competency on conditioning the effectiveness of leader’s authenticity (Wei et al., 2018), immoral behaviors (Mao et al., 2019), and empowerment (Cai et al., 2023), and authoritarian approach (Huang et al., 2023). Following this line of research and utilizing the interactional approach to leadership, we explore how leader competency moderates the influence of ethical leadership, which is also adding to the contingent view of leadership effectiveness. This analysis will add to the ongoing discussion on the moderating role of leader competency over different styles and behaviors of leadership, and make contributions to the interactive approach of studying leadership effectiveness.

When ethical leaders are considered competent, the social learning mechanism might be even more pronounced; that is, followers pay attention to, learn from, identify with, and accept the influence of an ethical leader who is competent. Learning from a competent leader means a higher possibility of future rewards, personal growth, and access to valuable resources. Moreover, competent leaders could have better upward exchange relationships with higher-level managers (Hollander, 1978). In this sense, competent ethical leaders can enhance group cohesiveness, esteem, and resources (Connelly et al., 2000; Mao et al., 2019). Followers believe that competent ethical leaders can provide valuable feedback, ample support, professional assistance, and credible guidance. Furthermore, because competency signals to followers that the future is bright with safe and ample resources, they develop engagement. On the contrary, when ethical leaders are not competent, their morality might be considered a weak expression of goodwill that will not lead to group success and prosperity. In this case, the effectiveness of ethical leadership might be less pronounced.

According to McAllister (1995), trust can be divided into affective and cognitive components. While the former relates to affiliation, empathy, and altruism, the latter relates to the objective evaluation of a leader’s competence, ability, integrity, and reliability. In this study, leader competency is deemed an important factor leading to followers’ cognitive trust of their leaders. In addition, cognitive trust reportedly might be the foundation of affective trust (Lewicki and Bunker, 1996). For followers to trust their leaders and accept vulnerability, leader competency is at least as important as ethicality. Even with ethical intention and behaviors, less competent leaders cannot lead the team to become effective and may even cause it to fail. In this case, it is unlikely that followers would trust a person who only has good intentions but is unable to lead competently. After all, leader competency is “an important influencing factor in determining the quality of the relationship that members form with their leader” (Byun et al., 2017, p. 1139), as suggested by interactive approach of leadership. Therefore, we propose the following hypothesis.

*Hypothesis 3: Leader competency moderates the relationship between ethical leadership and work engagement (hypothesis 3a) and between ethical leadership and trust in the leader (hypothesis 3b) such that the relationships are stronger when leader competency is high.*

**Figure 1:**
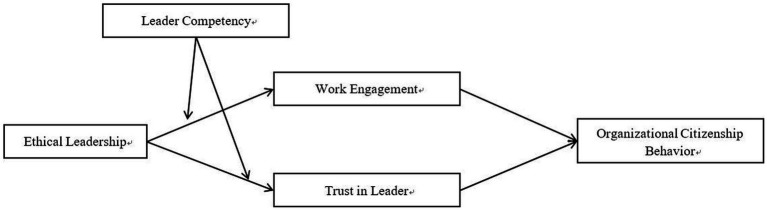
Hypothesized model.

By combining Hypotheses 2 and 3, we further present a moderated mediation model (Preacher et al., 2007), in which the influence of ethical leadership on OCB through work engagement and trust in the leader is moderated by leader competency (see Figure 1). Accordingly, we propose the following hypotheses.

*Hypothesis 4: Leader competency moderates the indirect relationship between ethical leadership and OCB through work engagement (hypothesis 4a) and trust in leader (hypothesis 4b) such that the indirect relationships are stronger when leader competency is high.*

## 3 Methods

### 3.1 Data and samples

To test the hypotheses, we surveyed customs officers in the Tianjin Customs of China. The duties of customs officers are highly professionalized in that their work involves customs inspection, clearance, and supervision; import and export statistics; protection of intellectual property; prevention of smuggling; and inspection and quarantine of contagion, among other responsibilities. Tianjin Customs of China has always attached great importance to ethical issues in their work and has carried out various professional ethics education themed practical activities. The purpose of providing professional ethics education to new customs officers before taking position is to continuously improve their professional ethics level, being honest, firm in their beliefs, fulfilling their duties, and handling affairs in accordance with the law. In December 2022, we distributed questionnaires to Chinese customs officers and implemented this study. We sent online survey links to 600 customs officers and obtained a sample of 329 valid responses (response rate = 54.83%) assessing their perceptions of ethical leadership, leader competency, organizational citizenship behavior, trust in leader and work engagement. We stated at the beginning of the questionnaire that the questionnaire is anonymous, and the data collected in the questionnaire will only be studied as a whole, and no personal information will be disclosed. Among the sample, 62% were male (*n* = 204); 71.7% had a bachelor’s degree (*n* = 236). Moreover, the average age was 37.75 years (SD = 8.56); average tenure was 14.84 years (SD = 9.52); and average tenure with the leader was 2.82 years (SD = 2.58). Our sample size is about 10 % of the total officers employed in Tianjin Customs and is quite representative of the demographics. However, our sample is slightly younger than the average age of the officers in Tianjin Customs (42 years old), which should be considered when interpreting the results.

### 3.2 Measures

#### 3.2.1 Ethical leadership

The 10-item Ethical Leadership Scale was used to measure ethical leadership (Brown et al., 2005). A sample item was “My supervisor disciplines employees who violate ethical standards.” The participants indicated their responses on a 5-point Likert-type scale, ranging from 1 = strongly disagree to 5 = strongly agree. The Cronbach’s alpha was 0.97.

#### 3.2.2 Leader competency

Leader competency was measured using a 13-item scale (Tett et al., 2000). A sample item is “My supervisor uses good judgment in resolving problems.” A 5-point Likert-type scale ranging from 1 = strongly disagree to 5 = strongly agree was used. The Cronbach’s alpha was 0.97.

#### 3.2.3 Work engagement

Work engagement was measured using three items (Schaufeli et al., 2019). A sample item is “At my work, I feel bursting with energy.” All items were assessed on a 5-point Likert-type scale ranging from 1 = strongly disagree to 5 = strongly agree. The Cronbach’s alpha was 0.93.

#### 3.2.4 Trust in the leader

Five items were used to measure trust in the leader (McAllister, 1995). A sample item was “If I shared my problems with my supervisor, I know (s)he would respond constructively and caringly.” The responses were evaluated on a 7-point Likert-type scale (1 = strongly disagree, 7 = strongly agree). The Cronbach’s alpha was 0.94.

#### 3.2.5 OCB

A 6-item scale was used to measure OCB (Van Dyne and LePine, 1998). A sample item was “I help others who have heavy workloads.” A 7-point Likert-type scale (1 = strongly disagree, 7 = strongly agree) was used. The Cronbach’s alpha was 0.91.

#### 3.2.6 Control variables

The respondents’ gender (1 = male, 2 = female), age (in years); education level (1 = three-year college degree and below, 2 = bachelor’s degree, 3 = master’s degree, and 4 = doctorate); tenure (in years); and tenure with the leader (in years) were included as controls, as they might be related to OCB, engagement, and trust.

## 4 Results

We use Mplus 8.3, SPSS 26.0 to perform statistical analysis. Hayes’ PROCESS macro for SPSS is used to test for mediation, moderation, and moderated mediation because of its widely accepted usage in these analyses.

### 4.1 Measurement model

Confirmatory factor analyses were used to test for convergent and discriminant validity (Table 1). The results indicated that the hypothesized five-factor model fitted the data well [*χ*^2^ = 1951.41, *df* = 619, comparative fit index (CFI) = 0.91, Tucker–Lewis index (TLI) = 0.91, root mean square error of approximation (RMSEA) = 0.08, and standardized root mean square residual (SRMR) = 0.03]. In addition, the factor loadings were all above 0.6 (0.62–0.94) and significant at the 0.001 level, providing evidence of convergent validity (Hu and Bentler, 1999). We also compared the proposed five-factor model to five alternative models to test for discriminant validity. The chi-square difference test and fit indexes revealed that the hypothesized five-factor model had the best fit. In addition, the average variance extracted (AVE) values for ethical leadership was 0.77; for leadership competence, 0.65; for trust in leader, 0.76; for work engagement, 0.78; and for OCB, 0.84. Moreover, the maximum shared variance values were smaller than the AVE values for the respective variables, and the average of the shared squared variances were smaller than the AVE values. Furthermore, the square roots of the AVE values were greater than the inter-construct correlations (Fornell and Larcker, 1981). These results indicate that the five-factor model achieves sufficient discriminant validity.

**Table 1:** Measurement model tests results.

		χ^2^	df	χ^2^/df	CFI	TLI	RMSEA	SRMR
1. Hypothesized 5-factor model	EL, LC, TL, WE, OCB	1951.41	619	3.15	0.91	0.91	0.08	0.03
2. Alternative 4-factor model	EL, LC + TL, WE, OCB	2768.95	623	4.45	0.86	0.85	0.10	0.07
3. Alternative 4-factor model	EL, LC + WE, TL, OCB	2709.11	623	4.35	0.87	0.86	0.10	0.07
4. Alternative 3-factor model	EL, LC + TL + WE, OCB	3467.17	626	5.54	0.82	0.81	0.12	0.08
5. Alternative 2-factor model	EL + LC + TL + WE, OCB	4212.50	628	6.71	0.77	0.76	0.14	0.07
6. Alternative 1-factor model	EL + TL + WE+LC + OCB	5356.37	629	8.52	0.70	0.68	0.15	0.10

EL, Ethical leadership; LC, Leader competency; TL, Trust in leader; WE, Work engagement; OCB, Organizational citizenship behavior; RMSEA, root mean square error of approximation, SRMR, standardized root mean square residual; CFI, comparative fit index; TLI, Tucker-Lewis index.

### 4.2 Hypotheses testing

Table 2 displays the descriptive statistics and correlations among the examined variables. The correlation patterns are consistent with our hypotheses.

**Table 2:** Means, standard deviations, and correlations among variables.

	Mean	SD	1	2	3	4	5	6	7	8	9	10
1. Gender	1.38	0.48	–									
2. Age	37.75	8.56	−0.07	–								
3. Education	2.25	0.48	0.02	−0.04	–							
4. Tenure	14.84	9.52	−0.05	0.90***	−0.10*	–						
5. Tenure with leader	2.82	2.58	0.04	0.15***	0.12*	0.17**						
6. Ethical leadership	3.82	0.95	0.07	−0.14**	0.00	−0.13*	0.00	(0.88)				
7. Leader competency	3.83	0.93	−0.01	−0.10*	0.00	−0.08	0.04	0.71***	(0.81)			
8. Trust in leader	4.64	1.48	−0.06	−0.01	−0.05	0.00	0.08	0.54***	0.53***	(0.87)		
9. Work engagement	3.46	1.05	−0.06	0.00	−0.02	0.02	0.10	0.50***	0.47***	0.50***	(0.88)	
10. OCB	5.61	1.09	−0.08	0.08*	−0.03	0.12*	0.12*	0.33***	0.32***	0.38***	0.43***	(0.92)

*N* = 329. Square roots of the AVE are values in parentheses along the diagonal.*p < 0.05, **p < 0.01, ***p < 0.001.

As shown in Table 3, ethical leadership is positively related to OCB (*β* = 0.48, *p* < 0.001, Model 3). Hypothesis 1 is thus supported. Further, Model 1 shows that ethical leadership is positively related to work engagement (*β* = 0.69, *p* < 0.001). Meanwhile, Model 2 suggests that ethical leadership is significantly positively related to trust in the leader (*β* = 0.75, *p* < 0.001). In Model 4, the effect of ethical leadership on OCB is not significant when including work engagement and trust in the leader in the model (*β* = 0.10, *p* > 0.05), while both the effects of work engagement (*β* = 0.33, *p* < 0.001) and trust in the leader (*β* = 0.21, *p* < 0.001) are significant. We used bootstrapping method to test for the significance of these indirect effects. The indirect effect through work engagement based on 5,000 bootstrapped samples is 0.31, with a 95% bias corrected confidence interval of [0.19, 0.43], excluding 0; the indirect effect through trust in the leader based on 5,000 bootstrapped samples is 0.28, with a 95% bias corrected confidence interval of [0.14, 0.43], excluding 0. Hypotheses 2a and 2b are thus supported.

**Table 3:** Test results for main and mediation effects.

	Work engagement	Trust in leader	OCB	
	Model 1	Model 2	Model 3	Model 4
Gender	−0.09 (0.09)	−0.11** (0.11)	−0.08 (0.11)	−0.03 (0.10)
Age	0.32 (0.03)	0.09 (0.03)	−0.30 (0.03)	−0.43 (0.03)
Education	−0.04 (0.10)	−0.06 (0.12)	0.04 (0.12)	0.06 (0.11)
Tenure	−0.23 (0.02)	−0.01 (0.03)	0.47 (0.03)	0.55*** (0.03)
Tenure with leader	0.09*** (0.02)	0.07 (0.02)	0.10 (0.02)	0.05 (0.02)
Ethical leadership	0.69*** (0.05)	0.75*** (0.06)	0.48*** (0.06)	0.10 (0.09)
Work engagement				0.33*** (0.07)
Trust in leader				0.21*** (0.05)
*R* ^2^	0.48	0.57	0.26	0.36
∆*R*^2^				0.10***
*F*	49.23***	70.50***	19.41***	22.68***

*N* = 329. Standardized coefficients are reported. Values in parentheses were standard error estimates.*p < 0.05, **p < 0.01, ***p < 0.001.

**Figure 2:**
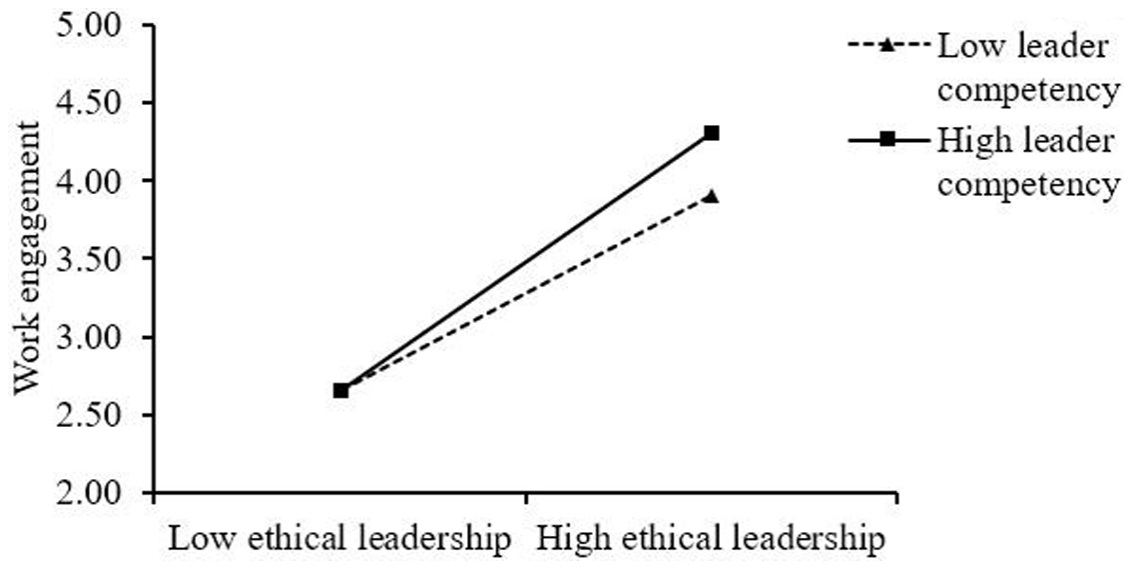
Interactive effects of ethical leadership and leader competency on work engagement.

**Figure 3:**
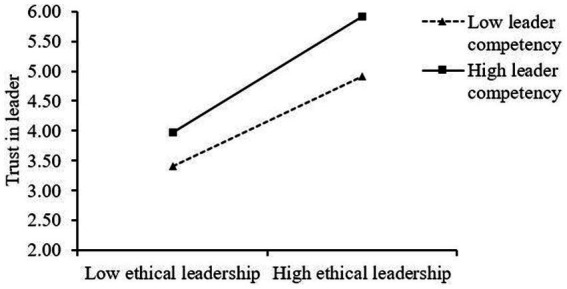
Interactive effects of ethical leadership and leader competency on trust in leader.

As Table 4 denotes, ethical leadership and leader competency interactively influence work engagement (*β* = 0.68, *p* < 0.01, Model 6) and trust in the leader (*β* = 0.54, *p* < 0.01, Model 8). To confirm the direction of these interactions, simple slopes were plotted at one standard deviation above and below the mean of leader competency. As Figure 2 illustrates, the slope of the relationship between ethical leadership and work engagement is steeper (simple slope = 0.86, *p* < 0.001) for the high leader-competency group than the low leader-competency group (simple slope = 0.66, *p* < 0.001). In Figure 3, the slope of the relationship between ethical leadership and trust in the leader is steeper for the high leader-competency group (simple slope = 1.02, *p* < 0.001) than the low leader-competency group (simple slope = 0.79, *p* < 0.001). Therefore, Hypotheses 3a and 3b are supported.

**Table 4:** Moderator effect test results.

	Work engagement		Trust in leader	
	Model 5	Model 6	Model 7	Model 8
Gender	−0.09 (0.09)	−0.08 (0.09)	−0.09 (0.11)	−0.09 (0.11)
Age	0.31 (0.03)	0.35 (0.03)	0.07 (0.03)	0.10 (0.03)
Education	−0.04 (0.10)	−0.05 (0.10)	−0.06 (0.12)	−0.07 (0.12)
Tenure	−0.22 (0.02)	−0.24 (0.02)	0.00 (0.03)	−0.01 (0.03)
Tenure with leader	0.09* (0.02)	0.08* (0.02)	0.06 (0.02)	0.06 (0.02)
Ethical leadership	0.63*** (0.09)	0.30* (0.15)	0.54*** (0.12)	0.27* (0.19)
Leader competency	0.07 (0.09)	−0.29* (0.16)	0.24** (0.12)	.-0.03 (0.20)
Ethical leadership* Leader competency		0.68** (0.04)		0.54** (0.05)
*R* ^2^	0.47	0.49	0.58	0.59
∆*R*^2^		0.02**		0.01**
*F*	42.23***	39.13***	63.84***	57.90***

*N* = 329. Standardized coefficients are reported. Values in parentheses were standard error estimates.*p < 0.05, **p < 0.01, ***p < 0.001.

As Table 5 denotes, for the indirect effect through work engagement, at a low level of leader competency, the indirect effect is 0.27, and the bootstrap confidence interval for the indirect effect does not include zero [0.15, 0.40]. At a high level of leader competency, the indirect effect is 0.35, and the bootstrap confidence interval for the indirect effect does not include zero [0.21, 0.51]. The index of the moderated mediation is significant (index = 0.05), and the bootstrap confidence interval does not include zero [0.02, 0.09]. These results support Hypothesis 4a. For the indirect effect through trust in the leader, at a low level of leader competency, the indirect effect is 0.19, and the bootstrap confidence interval for the indirect effect does not include zero [0.08, 0.34]. At a high level of leader competency, the indirect effect is 0.24, and the bootstrap confidence interval for the indirect effect does not include zero [0.10, 0.42]. The index of the moderated mediation is significant (index = 0.03), and the bootstrap confidence interval does not include zero [0.00, 0.06]. These results support Hypothesis 4b.

**Table 5:** Moderated mediation results across leader competency levels.

Ethical leadership - > work engagement - > OCB				
Indirect effect		SE	LL95%CI	UL95%CI
Leader competency M-1 SD (2.89)	0.27	0.06	0.15	0.40
Leader competency M + 1 SD (4.76)	0.35	0.08	0.21	0.51
Index of moderated mediation	Index	SE	LL95%CI	UL95%CI
	0.05	0.02	0.02	0.09
Ethical leadership - > trust in leader - > OCB				
Indirect effect		SE	LL95%CI	UL95%CI
Leader competency M-1 SD (2.89)	0.19	0.07	0.08	0.34
Leader competency M + 1 SD (4.76)	0.24	0.08	0.10	0.42
Index of moderated mediation	Index	SE	LL95%CI	UL95%CI
	0.03	0.01	0.00	0.06

*N* = 329. Bootstrap sample size = 5,000. LL, lower limit; CI, confidence interval; UL, upper limit.

## 5 Discussion

This study investigated the linking mechanism underlying ethical leadership and OCB in a sample of Chinese customs officers. Grounded in social learning, social exchange, and interactive psychology theories, we focused on the mediating roles of work engagement and trust in leader, and the moderating role of leader competency. The results indicate that ethical leadership is related to OCB through the mediating roles of work engagement and trust in leader. Furthermore, leader competency moderates the influence of ethical leadership on work engagement and trust in leader, and thus accentuates the influence of ethical leadership on OCB through work engagement and trust in leader. These findings have important theoretical and practical implications.

### 5.1 Theoretical implications

First, although ethicality is highly emphasized in public administration leadership research, the main focus is still on how it influences the (un)ethical behaviors of public employees (Bashir and Hassan, 2020; Young et al., 2021; Kim, 2023). By highlighting the influence of ethical leadership on the OCB of professional customs officers, our findings reveal that ethical leadership in public organizations goes beyond influencing ethics-related outcomes, thus echoing the previous finding that ethical leadership is critical in public organizations (Bedi et al., 2015). While ethics is context-bound (Resick et al., 2006), the impact of different contexts such as industry, sector, culture, and country on the effectiveness of ethical leadership is worthy of attention (Den Hartog, 2015). The customs office is responsible for maintaining national security and facilitating and securing international trade, for which ethicality is crucial, especially when dealing with issues such as smuggling, infectious disease control, and managing fines and taxes. This is particularly the case in Tianjin Customs Office of China where ethic related training and practices are highly emphasized. Our result point out the importance of highlighting ethics and especially ethical leadership in the context of frontline public service delivery. As the uncertainty and collaborative nature of public service delivery is increasingly emphasized, public organizations such as customs offices need more OCB to be effective. Our finding—that ethical leadership promotes proactive behavior among public employees to go the extra mile to help their organization and colleagues—extends the nomological network of ethical leadership in the context of public administration and locates the antecedent of OCB from the perspective of public leadership.

Second, going beyond the focus on prosocial motivation (Wright et al., 2016), our mediation analysis simultaneously incorporates work engagement and trust in the leader. This aspect is important as we have demonstrated that ethical leadership can improve the relationship of public employees with the work and their leader (Mostafa and Abed El-Motalib, 2018; Mozumder, 2018; Asif et al., 2019), which are two important associations. Our finding suggests that the motivational function of ethical leadership is not limited to instilling values and morality in followers to promote ethical behaviors or prevent unethical ones but it can also improve important work relationships and further translate into public employees’ proactivity. Thus, ethical leadership is meaningful to public organizations beyond morality, that is, achieving effectiveness. More research is needed to examine whether ethical leadership is related to other forms of work relationships (beyond our choice of work engagement and trust in leader), including leader–member exchange, or even public employees’ relationships with citizens.

Third, we found that leader competency is an important contingent factor influencing how followers perceive and react to ethical leadership. This finding echoes the results of early research on the importance of both task and relationship orientation (Yukl, 2010), and those of the moderating role of leader competency over empowerment (Cai et al., 2023) and authenticity (Wei et al., 2018). Although leader competency is emphasized as the most important determinant of leader effectives (Hollander, 1978), its examination, especially in public administration, remains limited. Understanding professional bureaucrats’ emphases on both ethicality and competency, we apply the followers’ perspective by highlighting the importance of their perception of whether their leader has both ethicality and competency. In essence, we found that for followers to develop engagement with work and trust in the leader and display OCB, public leaders need to demonstrate ethicality and competency simultaneously. Future research should further investigate how leader competency might condition leader’s traits and behaviors. For example, it would be interesting to see whether leader competency can mitigate or exacerbate the negative influence of an immoral leadership approach such as abusive leadership. Moreover, although leader competency is recognized in the literature (Wei et al., 2018; Mao et al., 2019; Cai et al., 2023), knowledge on its effect process remains limited. Our finding that leader competency interacts with ethical leadership to influence engagement and trust in the leader extends this line of investigation. However, more research is needed on how leader competency influences the perception and behaviors of followers. For example, leader competency might be related to followers’ cognitive trust, while affective trust might relate more to leaders’ benevolence and integrity (McAllister, 1995).

### 5.2 Practical implications

First, our findings suggest that ethical leadership significantly improves public employees’ work engagement, trust in the leader, and OCB. Therefore, ethical leadership should be encouraged in public administration. When selecting leaders, organizations should examine their moral orientation and stage of moral development. In terms of leadership training and cultivation, organizations can improve leader ethicality through case studies, role modeling, rhetorical devices, and so on. Second, because work engagement and trust in the leader transmits the influence of ethical leadership and acts as antecedents of OCB, public employees’ engagement and trust levels should be constantly surveyed to monitor and encourage more OCB. Additional support, autonomy, and feedback should be provided to enhance the engagement and trust levels of public employees. Third, the moderating role of leader competency suggests that in the professional public administration context, it is important to select and develop competent leaders, as they are deemed important by followers. Leadership rhetoric in the public context should not only emphasize ethicality nor ignore competency.

### 5.3 Limitations and suggestions for future research

Several limitations must be considered when interpreting the results of this study. First, the cross-sectional data used here cannot infer causal relationships among variables. Future research can use an experimental design to validate our findings. Second, this study was carried out only in Tianjin Customs of China; hence, the generalizability of the findings might be limited. Future research can investigate other contexts such as non-profit organizations, voluntary organizations, and other industries to examine the links between leader ethicality and competency.

Third, common method variance (CMV) could be a problem in this study since all the variables are self-reported by the respondents (Podsakoff et al., 2003). To remedy this problem, we applied several design features in the survey including guaranteed anonymity and detailed cover letter to emphasize the academic nature of the study. The items related to dependent, mediator, moderator, and independent variables were separated in the questionnaire. We also used Harman’s one-factor test to access CMV. The result of an exploratory factor analysis using a principal components extraction indicated the presence of five factors with the first factor explaining only 30.67% of the variance while the five factors in the total explained 79.96% of the variance. There is also evidence suggesting that interaction effects cannot be the artifacts of CMV but rather be deflated by it (Siemsen et al., 2010). Since we have found interaction effect in this study, CMV might be less of a concern. The fact that CMV cannot be completely eliminated in this study invites more studies using multi-method approaches to investigate the relationships among our studied variables further.

Fourth, compared with studies on the relationship between ethical leadership and OCB in other countries, the moderating variables selected in this study are relatively unique. For example, Mostafa (2018) selected organizational identification as the moderating variable and used a sample of Egyptian banking industry employees. The results revealed that the positive relationship between ethical leadership and OCBs was stronger for those lower in organizational identification. Sarwar et al. (2023) used high performance managerial practices (HPMPs) as a moderating variable, taking employees of private commercial banks in (Pakistan) as a sample, and the results showed that HPMPs enhance OCB by increasing the impact of ethical leadership on employee psychological empowerment. Tan et al. (2019) used ethnic dissimilarity as a moderating variable and collected data in private universities in China, Malaysia and Thailand. The study found that the relationship between perceived organizational support and OCB is stronger in heterogeneous sample group. The requirements for leadership in Eastern culture not only lie in morality, but also emphasize on ability, that is, having both ability and political integrity is the standard for qualified leadership in an environment. Therefore, this study explores the strengthening effect of leader competency on ethical leadership, and the results support the hypothesis of this article. However, the sample of this study is limited to China. Asian countries such as Singapore, Japan, and South Korea have relatively similar cultures to China. Whether the findings of current study can be generalized to these countries is not clear and necessitates further examination. Therefore, future research can collect samples from other countries to provide new evidence for the relationship between culture and morality.

## 6 Conclusion

Public service delivery should be conducted in both prosocial and professional manner. While ethical leadership reflects the morality and appropriateness of leaders’ actions, leader competency reflects their intelligence and ability to achieve desired goals. To successfully lead an organization, especially public sector organizations, leaders should simultaneously display ethicality and competency through their behaviors.

While ethical leadership is emphasized in the context of public administration, we illustrate in this study that leader competency also plays a vital role. This study uses survey data from 329 Chinese customs officials to investigate the impact of ethical leadership on customs officials’ OCB, focusing on the mediating roles of work engagement and trust in leader, and the moderating role of leader competency. When leading in an ethical manner, leaders can motivate follower to display OCB through developing both their engagement with the work itself and their trust in leaders. Furthermore, when ethical leadership behaviors are accompanied by leader competency, the beneficial influences of ethical leadership are accentuated. By developing and testing a moderated mediation model, this study depicts a nuanced interactive mechanism that emphasizes the duality of ethicality and competency in public leadership research. In this regard, in order to motive employees to act in proactive ways, public sector organizations should select and develop leaders to be both ethical and competent. Public sector organizations cannot simply emphasize the high moral standards of leaders while neglecting leader’s competency. Meanwhile, only emphasizing leader competency while neglecting ethicality is proven to be problematic. The dual appearance of both ethicality and competency is important, especially in the public domain.

## Data availability statement

The raw data supporting the conclusions of this article will be made available by the authors, without undue reservation.

## Ethics statement

The studies involving human participants were reviewed and approved by the Renmin University of China. The patients/participants provided their written informed consent to participate in this study.

## Author contributions

ZF led the literature review, research design, and manuscript drafting work. MH made contributions in data analysis and manuscript drafting work. YB made contributions in research design and manuscript drafting work. QS made contributions in data collection. All authors contributed to the article and approved the submitted version.

## Appendix

Table A1 Measurement items.

**Table:** 

Variable names	Items
Ethical leadership	My supervisor conducts his/her personal life in an ethical manner. My supervisor defines success not just by results but also the way that they are obtained. My supervisor listens to what employees have to say. My supervisor disciplines employees who violate ethical standards. My supervisor makes fair and balanced decisions. My supervisor can be trusted. My supervisor discusses business ethics or values with employees. My supervisor sets an example of how to do things the right way in terms of ethics. My supervisor has the best interests of employees in mind. When making decisions, my supervisor asks “what is the right thing to do?”
Leader competency	1.Problem Awareness: Perceives situations that may require action to promote organizational success. 2.Decision Making: Uses good judgment in resolving problems. 3.Directing: Clearly specifies to subordinates what needs to be done. 4.Decision Delegation: Assigns true decision-making authority to qualified subordinates. 5.Short-term Planning: Prepares the steps needed to complete tasks before action is taken. 6.Strategic Planning: Develops long-term plans to keep the organization aligned with future demands. 7.Coordinating: Organizes the activities of subordinates and the allocation of resources. 8.Goal Setting: Identifies organizational work unit objectives and the methods for achieving them. 9.Monitoring: Compares current work unit progress to predetermined standards, objectives, and deadlines. 10.Motivating by Authority: Influences subordinates directly using rewards and/or punishments. 11.Motivating by Persuasion: Persuades others to achieve excellence for its own sake. 12.Team Building: Identifies and integrates distinct subordinate roles in a spirit of collaboration. 13.Productivity: Accomplishes goals set by self or others.
Work engagement	1.At my work, I feel bursting with energy. 2.I am enthusiastic about my job. 3.I am immersed in my work.
Trust in leader	1.We have a sharing relationship. We can both freely share our ideas, feelings, and hopes. 2.I can talk freely to this individual about difficulties I am having at work and know that (s)he will want to listen. 3.We would both feel a sense of loss if one of us was transferred and we could no longer work together. 4.If I shared my problems with this person, I know (s)he would respond constructively and caringly. 5.I would have to say that we have both made considerable emotional investments in our working relationship.
OCB	1.I help others who have heavy workloads. 2.I willingly help others who have work related problems. 3.I am always ready to lend a helping hand to those around me. 4.I take steps to try to prevent problems with other workers. 5.I am mindful of how my behavior affects other people’s jobs. 6.I do not abuse the rights of others.

## References

[ref1] Asif M. Qing M. Hwang J. Shi H. (2019). Ethical leadership, affective commitment, work engagement, and creativity: testing a multiple mediation approach. Sustainability 11:4489. doi: 10.3390/su11164489

[ref2] Bakker A. B. Demerouti E. (2014). “Job demands–resources theory” in Work and wellbeing: wellbeing: a complete reference gudie. eds. Chen P. Y. Cooper C. L. (Chichester: Wiley)

[ref3] Bandura A. (1991). “Social cognitive theory of moral thought and action” in Handbook of moral behavior and development. eds. Kurtines W. M. Gewwirtz J. L. (Hillsdale, Nj: Lawrence Erlbaum)

[ref4] Bashir M. Hassan S. (2020). The need for ethical leadership in combating corruption. Int. Rev. Adm. Sci. 86, 673–690. doi: 10.1177/0020852318825386

[ref5] Bedi A. Alpaslan C. M. Green S. (2015). A meta-analytic review of ethical leadership outcomes and moderators. J. Bus. Ethics 139, 1–20. doi: 10.1007/s10551-015-2625-1

[ref6] Belle N. Cantarelli P. (2019). Do ethical leadership, visibility, external regulation, and prosocial impact affect unethical behavior? Evidence from a laboratory and a field experiment. Rev. Public Person. Admin. 39, 349–371. doi: 10.1177/0734371X17721301

[ref7] Blau P. M. (1964). Exchange and power in social life New York, NY, Wiley.

[ref8] Breevaart K. Bakker A. B. Demerouti E. Derks D. (2016). Who takes the lead? A multi-source diary study on leadership, work engagement, and job performance. J. Organ. Behav. 37, 309–325. doi: 10.1002/job.2041

[ref9] Brehm J. Gates S. (1997). Working, shirking, and sabotage: bureaucratic response to a democratic public, Ann Arbor University of Michigan Press.

[ref10] Brown M. E. (2007). Misconceptions of ethical leadership: how to avoid potential pitfalls. Organ. Dyn. 36, 140–155. doi: 10.1016/j.orgdyn.2007.03.003

[ref11] Brown M. E. Treviño L. K. (2006). Ethical leadership: a review and future directions. Leadersh. Q. 17, 595–616. doi: 10.1016/j.leaqua.2006.10.004

[ref12] Brown M. E. Treviño L. K. Harrison D. A. (2005). Ethical leadership: a social learning perspective for construct development and testing. Organ. Behav. Hum. Decis. Process. 97, 117–134. doi: 10.1016/j.obhdp.2005.03.002

[ref13] Bundgaard L. Jacobsen C. B. Jensen U. T. (2021). “Leadership in the public sector: concepts, context and outlooks” in Research handbook on Hrm in the public sector. eds. Steijn B. Knies E. (Cheltenham: Edwards Elgar Publishing Limited)

[ref14] Burke C. S. Sims D. E. Lazzara E. H. Salas E. (2007). Trust in leadership: a multi-level review and integration. Leadership Q. 18, 606–632. doi: 10.1016/j.leaqua.2007.09.006

[ref15] Byun G. Dai Y. Lee S. Kang S. (2017). Leader trust, competence, Lmx, and member performance: a moderated mediation framework. Psychol. Rep. 120, 1137–1159. doi: 10.1177/0033294117716465, PMID: 28649921

[ref16] Cai Y. Zhou C. Li J. Sun X. (2023). Leaders’ competence matters in empowerment: implications on subordinates’ relational energy and task performance. Eur. J. Work Organ. Psy. 32, 389–401. doi: 10.1080/1359432X.2022.2161370

[ref17] Campbell J. W. Im T. (2016). Psm and turnover intention in public organizations: does change-oriented organizational citizenship behavior play a role? Rev. Publ. Pers. Admin. 36, 323–346. doi: 10.1177/0734371X14567366

[ref18] Chen D. Zhang Y. Ahmad A. B. Liu B. (2023). How to fuel public employees’ change-oriented organizational citizenship behavior: a two-wave moderated mediation study. Rev. Publ. Pers. Admin. 43, 185–208. doi: 10.1177/0734371X211052675

[ref19] Connelly M. S. Gilbert J. A. Zaccaro S. J. Threlfall K. V. Marks M. A. Mumford M. D. (2000). Exploring the relationship of leadership skills and knowledge to leader performance. Leadersh. Q. 11, 65–86. doi: 10.1016/S1048-9843(99)00043-0

[ref20] Cropanzano R. Mitchell M. S. (2005). Social exchange theory: an interdisciplinary review. J. Manag. 31, 874–900. doi: 10.1177/0149206305279602

[ref21] Davis T. R. V. Luthans F. (1980). A social learning approach to organizational behavior. Acad. Manag. Rev. 5, 281–290. doi: 10.2307/257438

[ref22] Den Hartog D. N. (2015). Ethical leadership. Annu. Rev. Organ. Psychol. Organ. Behav. 2, 409–434. doi: 10.1146/annurev-orgpsych-032414-111237

[ref23] Dirks K. T. Ferrin D. L. (2002). Trust in leadership: Meta-analytic findings and implications for research and practice. J. Appl. Psychol. 87, 611–628. doi: 10.1037/0021-9010.87.4.611, PMID: 12184567

[ref24] Erickson A. L. (2006). Ethical leadership and the public trust. Publ. Manag. 35:62.

[ref25] Fehr R. Kai C. Y. Dang C. (2015). Moralized leadership: the construction and consequences of ethical leader perceptions. Acad. Manag. Rev. 40, 182–209. doi: 10.5465/amr.2013.0358

[ref26] Fiske S. T. Cuddy A. J. C. Glick P. (2007). Universal dimensions of social cognition: warmth and competence. Trends Cogn. Sci. 11, 77–83. doi: 10.1016/j.tics.2006.11.005, PMID: 17188552

[ref27] Fornell C. Larcker D. F. (1981). Evaluating structural equation models with unobservable variables and measurement error. J. Mark. Res. 18, 39–50. doi: 10.1177/002224378101800104

[ref28] Frey B. S. Palacios-Huerta I. (1997). Not just for the money: an economic theory of personal motivation. Cheltenham, UK: Edward Elgar Pub.

[ref29] Hassan S. (2015). The importance of ethical leadership and personal control in promoting improvement-centered voice among government employees. J. Publ. Admin. Res. Theory 25, 697–719. doi: 10.1093/jopart/muu055

[ref30] Hassan S. Mahsud R. Yukl G. Prussia G. E. (2013). Ethical and empowering leadership and leader effectiveness. J. Manag. Psychol. 28, 133–146. doi: 10.1108/02683941311300252

[ref31] Hassan S. Wright B. E. Yukl G. (2014). Does ethical leadership matter in government? Effects on organizational commitment, absenteeism, and willingness to report ethical problems. Public Adm. Rev. 74, 333–343. doi: 10.1111/puar.12216

[ref32] Hoch J. E. Bommer W. H. Dulebohn J. H. Wu D. (2018). Do ethical, authentic, and servant leadership explain variance above and beyond transformational leadership? A meta-analysis. J. Manag. 44, 501–529. doi: 10.1177/0149206316665461

[ref33] Hollander E. P. (1978). Leadership dynamics: a practical guide to effective relationships. New York: The Free Press.

[ref34] Hu L.-T. Bentler P. M. (1999). Cutoff criteria for fit indexes in covariance structure analysis: conventional criteria versus new alternatives. Struct. Equ. Model. Multidiscip. J. 6, 1–55. doi: 10.1080/10705519909540118

[ref35] Huang Q. Zhang K. Wang Y. Bodla A. A. Zhu D. (2023). When is authoritarian leadership less detrimental? The role of leader capability. Int. J. Environ. Res. Public Health 20:707. doi: 10.3390/ijerph20010707 PMC9819526 36613043

[ref36] Justis R. T. Kedia B. Stephens D. B. (1978). The effect of position power and perceived task competence on trainer effectivenss: a partial utilization of Fiedler's contingency model of leadership. Pers. Psychol. 31, 83–93. doi: 10.1111/j.1744-6570.1978.tb02111.x

[ref37] Kacmar K. M. Bachrach D. G. (2011). Fostering good citizenship through ethical leadership: exploring the moderating role of gender and organizational politics. J. Appl. Psychol. 96, 633–642. doi: 10.1037/a0021872 21142344

[ref38] Kaptein M. Huberts L. E. O. Avelino S. Lasthuizen K. (2005). Demonstrating ethical leadership by measuring ethics: a survey of U.S. public servants. Publ. Integr. 7, 299–311. doi: 10.1080/10999922.2005.11051286

[ref39] Kim J. (2023). Ethical leadership and program to reduce unethical behaviour among public employees, Public Manag. Rev. 25, 1333–1347. doi: 10.1080/14719037.2021.2015185

[ref40] Lewicki R. J. Bunker B. B. (1996). “Developing and maintaining trust in work relationships” in Trust in organizations: frontiers of theory and research. (eds) Roderick M. Kramer and Tom R. Tyler, 114–139.

[ref41] Li C. Dong Y. Wu C.-H. Brown M. E. Sun L.-Y. (2022). Appreciation that inspires: the impact of leader trait gratitude on team innovation. J. Organ. Behav. 43, 693–708. doi: 10.1002/job.2577

[ref42] Lin C.-S. Jin M. Huang P.-C. Xiao R. (2023). Does it take two to tango? The joint role of high-performance work systems and ethical leadership. J. Bus. Res. 156:113536. doi: 10.1016/j.jbusres.2022.113536

[ref43] Lu X. (2014). Ethical leadership and organizational citizenship behavior: the mediating roles of cognitive and affective trust. Soc. Behav. Personal. Int. J. 42, 379–389. doi: 10.2224/sbp.2014.42.3.379

[ref44] Lu X. Guy M. E. (2014). How emotional labor and ethical leadership affect job engagement for Chinese public servants. Publ. Pers. Manag. 43, 3–24. doi: 10.1177/0091026013512278

[ref45] Mansour M. Aman N. Al-Ghazali B. M. Shah S. H. A. (2022). Perceived corporate social responsibility, ethical leadership, and moral reflectiveness impact on pro-environmental behavior among employees of small and medium enterprises: a double-mediation model. Front. Psychol. 13:967859. doi: 10.3389/fpsyg.2022.967859, PMID: 36507031 PMC9727833

[ref46] Mao J.-Y. Chiang J. T.-J. Chen L. Wu Y. Wang J. (2019). Feeling safe? A conservation of resources perspective examining the interactive effect of leader competence and leader self-serving behaviour on team performance. J. Occup. Organ. Psychol. 92, 52–73. doi: 10.1111/joop.12233

[ref47] McAllister D. J. (1995). Affect-and cognition-based trust as foundations for interpersonal cooperation in organizations. Acad. Manag. J. 38, 24–59. doi: 10.2307/256727

[ref48] Mohsin M. Zhu Q. Wang X. Naseem S. Nazam M. (2021). The empirical investigation between ethical leadership and knowledge-hiding behavior in financial service sector: a moderated-mediated model. Front. Psychol. 12:798631. doi: 10.3389/fpsyg.2021.798631, PMID: 34975699 PMC8716561

[ref49] Molines M. Mifsud M. El Akremi A. Perrier A. (2022). Motivated to serve: a regulatory perspective on public service motivation and organizational citizenship behavior. Public Adm. Rev. 82, 102–116. doi: 10.1111/puar.13445

[ref50] Moon K.-K. Jung C. (2018). Management representativeness, ethical leadership, and employee job satisfaction in the US federal government. Publ. Pers. Manag. 3, 265–286. doi: 10.1177/0091026018767480

[ref51] Mostafa A. M. S. (2018). Ethical leadership and organizational citizenship behaviours: the moderating role of organizational identification. Eur. J. Work Organ. Psychol. 27, 441–449. doi: 10.1080/1359432X.2018.1470088

[ref52] Mostafa A. M. S. Abed El-Motalib E. A. (2018). Ethical leadership, work meaningfulness, and work engagement in the public sector. Rev. Public Pers. Admin. 40:0734371X18790628. doi: 10.1177/0734371X18790628

[ref53] Mostafa A. M. S. Farley S. Zaharie M. (2020). Examining the boundaries of ethical leadership: the harmful effect of co-worker social undermining on disengagement and employee attitudes. J. Bus. Ethics 174, 355–368. doi: 10.1007/s10551-020-04586-2

[ref54] Mozumder N. A. (2018). A multilevel trust-based model of ethical public leadership. J. Bus. Ethics. 153, 167–184. doi: 10.1007/s10551-016-3341-1

[ref55] Ng T. W. H. Feldman D. C. (2015). Ethical leadership: Meta-analytic evidence of criterion-related and incremental validity. J. Appl. Psychol. 100, 948–965. doi: 10.1037/a0038246, PMID: 25420055

[ref56] Niskanen W. A. (1971). Bureaucracy and representative government, Chicago, Aldine-Aherton.

[ref57] Organ D. W. (1988). Organizational citizenship behavior: the good soldier syndrome, Lexington, ma England, Lexington Books/D.C Heath and Com: Lexington, MA.

[ref58] Perry J. L. Vandenabeele W. (2015). Public service motivation research: achievements, challenges, and future directions. Public Adm. Rev. 75, 692–699. doi: 10.1111/puar.12430

[ref59] Perry J. L. Wise L. R. (1990). The motivational bases of public service. Public Adm. Rev. 50, 367–373. doi: 10.2307/976618

[ref60] Podsakoff P. M. Mackenzie S. B. Lee J.-Y. Podsakoff N. P. (2003). Common method biases in behavioral research: a critical review of the literature and recommended remedies. J. Appl. Psychol. 88, 879–903. doi: 10.1037/0021-9010.88.5.879, PMID: 14516251

[ref61] Podsakoff P. M. Todor W. D. Schuler R. S. (1983). Leader expertise as a moderator of the effects of instrumental and supportive leader behaviors. J. Manag. 9, 173–185. doi: 10.1177/014920638300900208

[ref62] Potipiroon W. Faerman S. (2016). What difference do ethical leaders make? Exploring the mediating role of interpersonal justice and the moderating role of public service motivation. Int. Public Manag. J. 19, 171–207. doi: 10.1080/10967494.2016.1141813

[ref63] Preacher K. J. Rucker D. D. Hayes A. F. (2007). Addressing moderated mediation hypotheses: theory, methods, and prescriptions. Multivar. Behav. Res. 42, 185–227. doi: 10.1080/00273170701341316 26821081

[ref64] Price K. H. Garland H. (1981). Compliance with a leader's suggestions as a function of perceived leader/member competence and potential reciprocity. J. Appl. Psychol. 66, 329–336. doi: 10.1037/0021-9010.66.3.329

[ref65] Resick C. J. Hanges P. J. Dickson M. W. Mitchelson J. K. (2006). A cross-cultural examination of the endorsement of ethical leadership. J. Bus. Ethics 63, 345–359. doi: 10.1007/s10551-005-3242-1

[ref66] Ritz A. Giauque D. Varone F. Anderfuhren-Biget S. (2014). From leadership to citizenship behavior in public organizations: when values matter. Rev. Publ. Pers. Admin. 34, 128–152. doi: 10.1177/0734371X14521456

[ref67] Rousseau D. M. Sitkin S. B. Burt R. S. Camerer C. (1998). Not so different after all: a cross-discipline view of trust. Acad. Manag. Rev. 23, 393–404. doi: 10.5465/amr.1998.926617

[ref68] Sarwar N. Haider S. Akhtar M. H. Bakhsh K. (2023). Moderated-mediation between ethical leadership and organizational citizenship behavior: the role of psychological empowerment and high performance managerial practices. Manag. Res. Rev. 46, 649–666. doi: 10.1108/MRR-07-2021-0528

[ref69] Schaufeli W. B. Salanova M. González-Romá V. Bakker A. B. (2002). The measurement of engagement and burnout: a two sample confirmatory factor analytic approach. J. Happiness Stud. 3, 71–92. doi: 10.1023/A:1015630930326

[ref70] Schaufeli W. B. Shimazu A. Hakanen J. Salanova M. Witte H. D. (2019). An ultra-short measure for work engagement. Eur. J. Psychol. Assess. 35, 577–591. doi: 10.1027/1015-5759/a000430

[ref71] Siemsen E. Roth A. V. Oliveira P. (2010). Common method bias in regression models with linear, quadratic, and interaction effects. Organ. Res. Methods 13, 456–476. doi: 10.1177/1094428109351241

[ref72] Stein M. Schümann M. Vincent-Höper S. (2021). A conservation of resources view of the relationship between transformational leadership and emotional exhaustion: the role of extra effort and psychological detachment. Work Stress 35, 241–261. doi: 10.1080/02678373.2020.1832610

[ref73] Tan L. P. Yap C. S. Choong Y. O. Choe K. L. Rungruang P. Li Z. (2019). Ethical leadership, perceived organizational support and citizenship behaviors. Leadersh. Organ. Dev. J. 40, 877–897. doi: 10.1108/LODJ-04-2019-0160

[ref74] Tett R. P. Guterman H. A. Bleier A. Murphy P. J. (2000). Development and content validation of a “hyperdimensional” taxonomy of managerial competence. Hum. Perform. 13, 205–251. doi: 10.1207/S15327043HUP1303_1

[ref75] Tian G. Pu L. Ren H. (2021). Gender differences in the effect of workplace loneliness on organizational citizenship behaviors mediated by work engagement. Psychol. Res. Behav. Manag. 14, 1389–1398. doi: 10.2147/PRBM.S329959 34512049 PMC8427293

[ref76] Treviño L. K. Brown M. E. (2007). Ethical leadership: A developing construct. In Positive Organizational Behavior. (eds) D. Nelson, and C. L. Copper SAGE Publications Inc., 101–116.

[ref77] Van Dyne L. Lepine J. A. (1998). Helping and voice extra-role behaviors: evidence of construct and predictive validity. Acad. Manag. J. 41, 108–119. doi: 10.2307/256902

[ref78] Van Wart M. (2003). Public-sector leadership theory: an assessment. Public Adm. Rev. 63, 214–228. doi: 10.1111/1540-6210.00281

[ref79] Van Wart M. (2011). “Changing dynamics of administrative leadership” in The state of public administration: issues, challenges, and opportunities. eds. Menzel D. C. White H. L. (M. E. Sharpe: Armonk, NY)

[ref80] Van Wart M. (2012). Dynamics of leadership in public service: theory and practice, Armonk, NY M. E Sharpe.

[ref81] Van Wart M. (2013). Administrative leadership theory: a reassessment after 10 years. Public Adm. 91, 521–543. doi: 10.1111/padm.12017

[ref82] Wei F. Li Y. Zhang Y. Liu S. (2018). The interactive effect of authentic leadership and leader competency on followers’ job performance: the mediating role of work engagement. J. Bus. Ethics 153, 763–773. doi: 10.1007/s10551-016-3379-0

[ref83] Weinberg H. (2014). Ethical leadership in public service: a solid foundation for good government. Public Adm. Rev. 74, 344–345. doi: 10.1111/puar.12226

[ref84] Wright B. E. Hassan S. Park J. (2016). Does a public service ethic encourage ethical behavior? Public service motivation, ethical leadership and the willingness to report ethical problems. Public Adm. 94, 647–663. doi: 10.1111/padm.12248

[ref85] Xu J. Xie B. Chung B. (2019). Bridging the gap between affective well-being and organizational citizenship behavior: the role of work engagement and collectivist orientation. Int. J. Environ. Res. Public Health 16:4503. doi: 10.3390/ijerph16224503, PMID: 31731590 PMC6888382

[ref86] Young K. A. Hassan S. Hatmaker D. M. (2021). Towards understanding workplace incivility: gender, ethical leadership and personal control. Public Manag. Rev. 23, 31–52. doi: 10.1080/14719037.2019.1665701

[ref87] Yukl G. A. (2010). Leadership in organizations. Upper Saddle River, NJ, Prentice Hall.

[ref88] Zahari N. Kaliannan M. (2023). Antecedents of work engagement in the public sector: a systematic literature review. Rev. Publ. Pers. Admin. 43, 557–582. doi: 10.1177/0734371X221106792

[ref89] Zhang L. Farndale E. (2022). Workforce age profile effects on job resources, work engagement and organizational citizenship behavior. Pers. Rev. 51, 194–209. doi: 10.1108/PR-02-2020-0095

[ref90] Zhu W. Newman A. Miao Q. Hooke A. (2013). Revisiting the mediating role of trust in transformational leadership effects: do different types of trust make a difference? Leadersh. Q. 24, 94–105. doi: 10.1016/j.leaqua.2012.08.004

